# Zika virus impairs the development of blood vessels in a mouse model of congenital infection

**DOI:** 10.1038/s41598-018-31149-3

**Published:** 2018-08-24

**Authors:** P. P. Garcez, H. B. Stolp, S. Sravanam, R. R. Christoff, J. C. C. G. Ferreira, A. A. Dias, P. Pezzuto, L. M. Higa, J. Barbeito-Andrés, R. O. Ferreira, C. B V. Andrade, M. Siqueira, T. M. P. Santos, J. Drumond, A. Hoerder-Suabedissen, C. V. F. de Lima, F. Tovar-Moll, R. T. Lopes, L. Fragel-Madeira, R. Lent, T. M. Ortiga-Carvalho, J. Stipursky, M. Bellio, A. Tanuri, Z. Molnár

**Affiliations:** 1 Institute of Biomedical Sciences, Federal University of Rio de Janeiro, Rio de Janeiro, Brazil; 2 Department of Physiology, Anatomy and Genetics, University of Oxford, Oxford, UK; 3 Centre for the Developing Brain, Department of Perinatal Imaging & Health, King’s College London, London, UK; 4 Microbiology Institute Paulo de Góes, Federal University of Rio de Janeiro, Rio de Janeiro, Brazil; 5 Institute of Biology, Federal University of Rio de Janeiro, Rio de Janeiro, Brazil; 6 Institute of Biophysics Carlos Chagas Filho, Federal University of Rio de Janeiro, Rio de Janeiro, Brazil; 7 Nuclear Instrumentation Laboratory, Federal University of Rio de Janeiro, Rio de Janeiro, Brazil; 8 D’Or Institute for Research and Education (IDOR), Rio de Janeiro, Brazil; 9 Department of Neurobiology, Institute of Biology, Fluminense Federal University, Niterói, Brazil

## Abstract

Zika virus (ZIKV) is associated with brain development abnormalities such as primary microcephaly, a severe reduction in brain growth. Here we demonstrated *in vivo* the impact of congenital ZIKV infection in blood vessel development, a crucial step in organogenesis. ZIKV was injected intravenously in the pregnant type 2 interferon (IFN)-deficient mouse at embryonic day (E) 12.5. The embryos were collected at E15.5 and postnatal day (P)2. Immunohistochemistry for cortical progenitors and neuronal markers at E15.5 showed the reduction of both populations as a result of ZIKV infection. Using confocal 3D imaging, we found that ZIKV infected brain sections displayed a reduction in the vasculature density and vessel branching compared to mocks at E15.5; altogether, cortical vessels presented a comparatively immature pattern in the infected tissue. These impaired vascular patterns were also apparent in the placenta and retina. Moreover, proteomic analysis has shown that angiogenesis proteins are deregulated in the infected brains compared to controls. At P2, the cortical size and brain weight were reduced in comparison to mock-infected animals. In sum, our results indicate that ZIKV impairs angiogenesis in addition to neurogenesis during development. The vasculature defects represent a limitation for general brain growth but also could regulate neurogenesis directly.

## Introduction

Primary microcephaly is a severe brain malformation characterized by the reduction of the cephalic perimeter at birth. Microcephaly etiologies vary from genetic abnormalities to external factors such as the TORCHS pathogens (***T****oxoplasma gondii*, **o**thers, **R**ubella, **C**ytomegalovirus, ***H****erpes simplex* virus and **S**yphilis). Recently, Zika virus (ZIKV) has been associated with primary microcephaly^1^, and it is considered a new TORCHS factor^2^. ZIKV infects human neural progenitors and impairs the growth of 3D differentiating cultures, such as neurospheres and brain organoids^3–5^. Moreover, *in vitro*, ZIKV alters the cell cycle and induces apoptosis in neural progenitor cells^4,6^. Studies using mouse models showed brain growth impairment as a result of ZIKV congenital infection^7–9^. However, the cellular and molecular mechanisms that underlie the brain growth failure in ZIKV congenital syndrome remain to be fully understood.

We hypothesize that disruption of neurovascular development is one of the primary determinants of ZIKV congenital syndrome. The cortical vascular plexus development starts as early as E11 in the mouse and is essential for the delivery of nutrients and for regulating the expression of signaling molecules, impacting on neurogenesis^10,11^. The cortical blood supply develops from the ventricular and the pial plexi^12,13^. In the embryonic mouse brain, the formation of the vascular plexus is also partially driven by morphogenic factors such as Dlx1/2 and Pax6^14^ and ensure adequate coverage of the neurogenic zones. Disruption of the vascular network has previously been shown to alter neurogenesis and cortical patterning^12^.

Here, we describe the use of a congenital mouse model of ZIKV-induced microcephaly, to investigate the effects of ZIKV infection on neuro-vascular development. Infecting type 2 interferon (IFN)-deficient *(Ifng*^*−/−*^) pregnant mice with ZIKV at E12.5 resulted in reduced brain size at birth. At E15.5, neurogenesis was affected by ZIKV infection, radial glia and Ctip2 neuronal population are reduced compared to control animals. Moreover, blood vessels were found to be immature, with fewer branches and reduced area at E15.5; key angiogenesis proteins were also found to be deregulated in the infected cerebral cortex at this time. The results reported here suggest a mechanism through which ZIKV infection of endothelial cells may contribute to the Zika congenital syndrome-like abnormalities demonstrated in this mouse model.

## Results

### ZIKV Infection Reduces Brain Size and Cortical Thickness in P2 Mouse Pups

**Figure 1:**
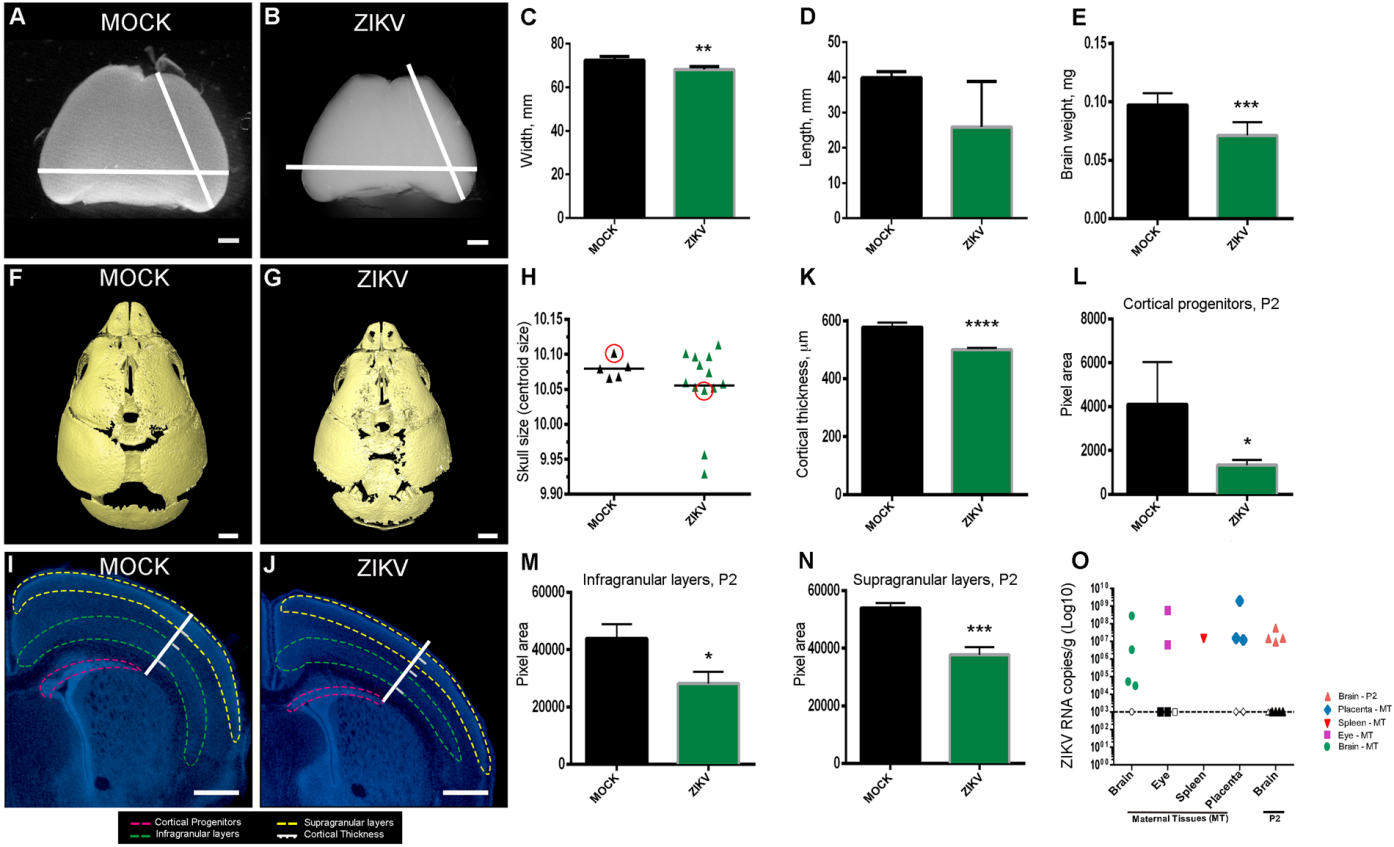
Reduced brain length, area and weight at P2 following ZIKV infection Light microscope image of P2 MOCK (**A**) or ZIKV infected (**B**) whole brain, showing reduced cortical length and lateral width (**C**) and length (**D**); (**E**) ZIKV infected brains show a significant decrease in brain weight compared to MOCK brains at P2. Brains were obtained from three litters, n = 12. Student’s t-test **p < 0.01 in C; ***p < 0.001 in E. CT reconstructed skulls of MOCK (**F**) and ZIKV (**G**) P2 pups injected IV at E12.5. Scale bar = 1 mm (**H**) Quantification of skull size obtained P2 animals from three litters, n = 18. Circles represent the skulls in F and G. DAPI stained sections of MOCK (**I**) and ZIKV infected (**J**) brains with white measurement bars for illustration of cortical thickness, with boundaries for normotypical white matter and cortical layer 4 marked. Cortical progenitor area is highlighted in pink, infragranular layers in green and supragranular, in yellow. (**K**) ZIKV brains show a significant decrease in cortical thickness compared to MOCK brains; Scale bars A, B = 1 mm; C, D = 500 µm. (**L**) ZIKV infected P2 brains show a significant decrease in thickness compared to MOCK brains in the cortical progenitor layers, infragranular layers (**M**) and supragranular layers (**N**). Brains were obtained from three litters, n = 12. Student’s t-test ****p < 0.0001 in K; *p < 0.05 in L and M; ***p < 0.001 in N. (**O**) ZIKV RNA was quantified by qRT-PCR from maternal brain, eye and spleen and P2 brain tissues collected 9 days post infection. Closed symbols indicate MOCK mice, open symbols indicate ZIKV infected mice and dotted line represents the limit of detection. Tissues were obtained from n = 5 adults and n = 7 pups.

ZIKV was injected intraveneouly (retro-orbital) in *Ifng*^*−/−*^ pregnant females at E12.5. At P2, the primary microcephaly-like syndrome was characterized by the smaller brain size, lower brain weight, and smaller skull size. Brain width and length was decreased in ZIKV infected brains compared to MOCK brains (Fig. 1A–D). There was a significant decrease in the weight of ZIKV infected brains (71.1 µg ± 3.7 µg, n = 9) compared to MOCK brains (100.5 µg ± 9.3 µg, n = 3) at P2 (p = 0.005; Fig. 1E). Similarly, skull size was reduced in ZIKV infected pups, although this effect showed considerable variability (Fig. 1F–H). Brain cortical thickness and cortical area were measured from DAPI stained sections at P2. The cortical thickness of ZIKV infected brains (501 µm ± 1.5 µm, n = 13, Fig. 1J) was significantly reduced compared to MOCK brains (583.3 µm ± 3.2 µm, n = 16, Fig. 1I). Statistical analysis showed that at P2, there was a decrease in parietal cortical wall area (Fig. 1K), cortical progenitor layers (Fig. 1L) and areas of cortical layers II and III (Fig. 1M), as well as V and VI (Fig. 1N) in ZIKV infected pups compared to MOCK pups of same age. To determine the ZIKV RNA presence in maternal and newborn tissue, we used qPCR and detected ZIKV in the brain, eye, spleen, and placenta of infected mothers and in the brain of newborns 9 days post-infection (Fig. 1O, open symbols), in contrast to MOCK undetermined detection (Fig. 1O, closed symbols, dotted line).

### ZIKV Infection at E12.5 Induces Defective Neurogenesis at E15.5

**Figure 2:**
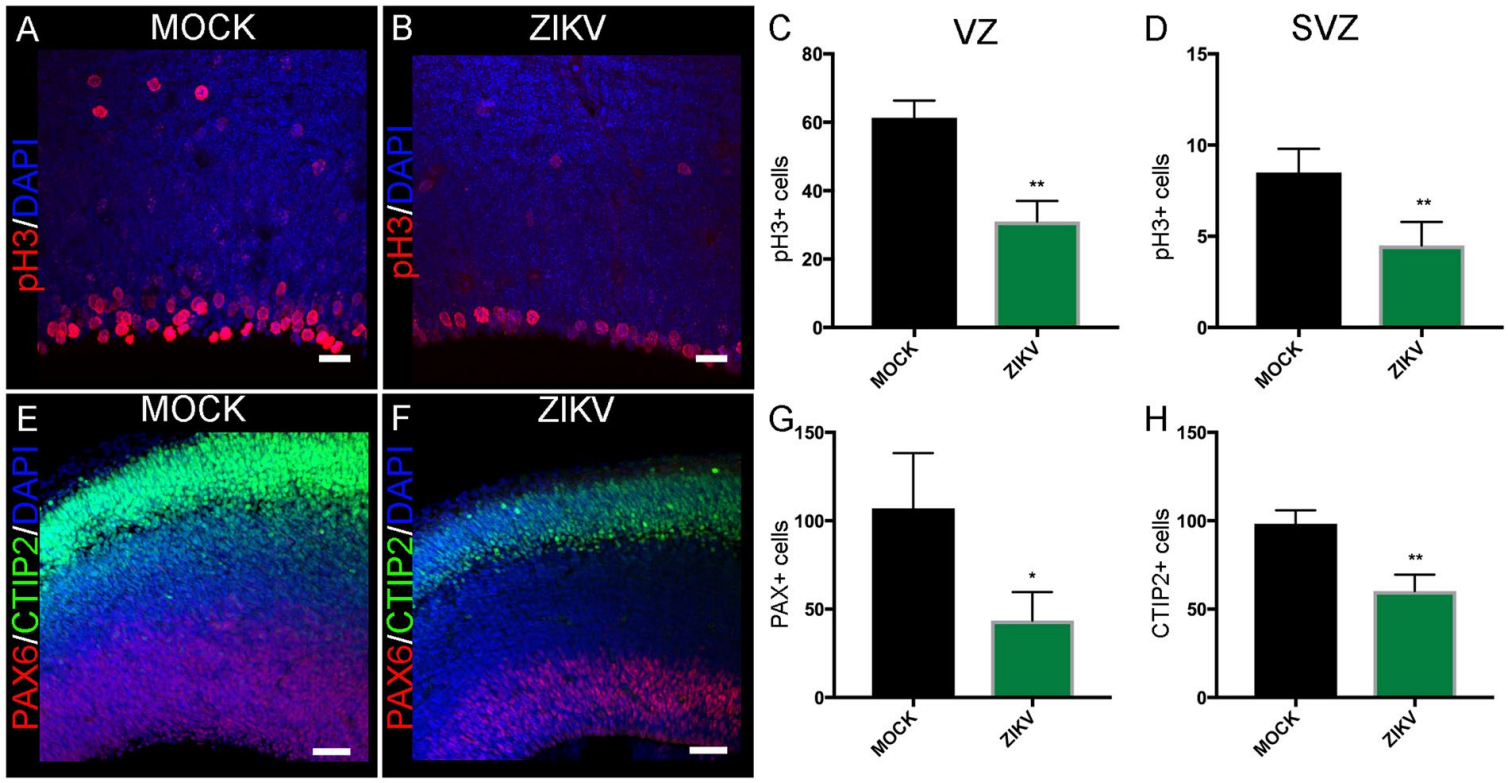
ZIKV infection was associated with reduced neurogenic proliferation Confocal images showing pH3 and DAPI co-stained in MOCK (**A**) and ZIKV (**B**) infected E15.5 brain sections. Data presented here as the mean ± SEM from sections prepared from three embryos obtained from three litters for each condition in this analysis and subsequent quantifications, unless stated otherwise. There is a significant decrease in pH3+ cells in VZ (**C**) and SVZ (**D**) of dorsal cortex in ZIKV infected brains compared to MOCK brains. MOCK, n = 184 cells; ZIKV, n = 93 cells, Student’s t-test **p < 0.01 in C and MOCK, n = 50 cells; ZIKV, n = 36 cells in D, Student’s t-test **p < 0.01. Confocal images showing Pax6, Ctip2, and DAPI co-stained in MOCK (**E**) and ZIKV (**F**) infected E15.5 brain sections. There is a significant decrease in Pax6+ (**G**) and Ctip2 (**H**) cells in ZIKV infected brains compared to MOCK brains at E15.5. MOCK, n = 321 cells; ZIKV, n = 174 cells, Student’s t-test *p < 0.05 in G and MOCK, n = 295 cells; ZIKV, n = 241 cells in H, Student’s t-test **p < 0.01. Scale bar = 20 μm.

To evaluate the impact of ZIKV congenital infection at E12.5 in neurogenesis, we harvested the brains three days after the infection. At E15.5, dividing cells in the VZ and SVZ were identified with the M-phase marker, phospho-histone H3 (pH3, Fig. 2). At E15.5, a significant reduction in pH3+ cell population was detected in the VZ in the cerebral cortex of ZIKV infected compared to MOCK animals (Fig. 2A–C). In the SVZ, the number of pH3+ cells in ZIKV infected brains was also reduced (Fig. 2A,B,D). There was an associated decrease in the Pax6 progenitor population in the VZ (43.5 ± 8.1 cells in infected brains compared to 107 ± 18.0 cells in MOCK brains, p = 0.016; Fig. 2E–G) as well as a reduction in the early-born CTIP2 positive neurons in the cortical plate (98.3 ± 4.4 cells in MOCK brains compared to 60.2 ± 4.6 cells in infected brains, p = 0.002; Fig. 2E,F,H). These results confirm that neurogenesis is impaired in the congenital ZIKV infection.

### ZIKV Infects Endothelial Cells and Reduces Cortical VZ Vascularization at E15.5

**Figure 3:**
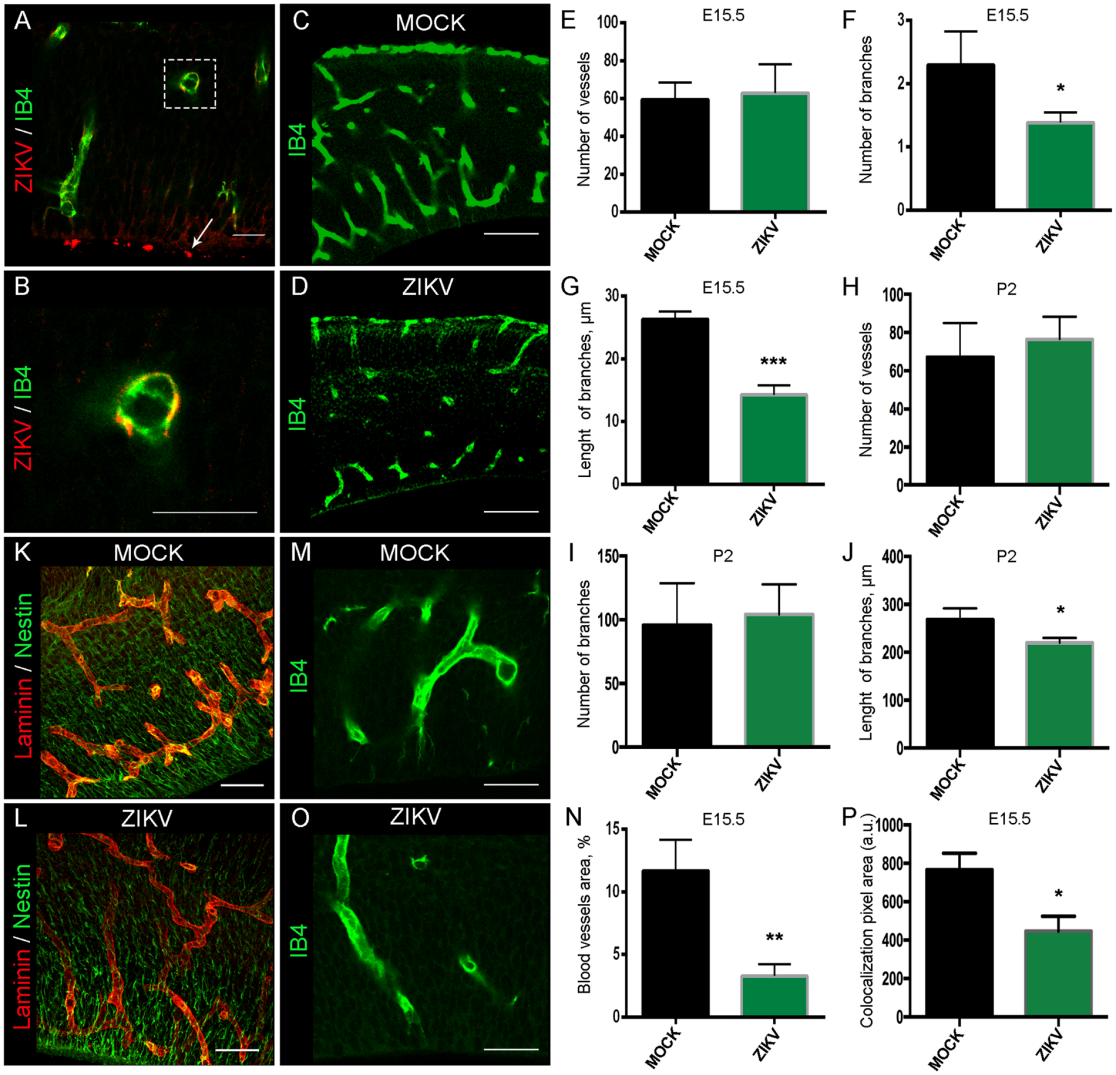
Immature blood vessel patterning in the VZ in ZIKV infected brains Confocal images of IB4 stained blood vessels and 4G2 flavivirus marker in ZIKV infected brain section at E15.5 (**A**) and higher magnification in (**B**) Arrow points to the ventricular zone. Confocal images of IB4 stained blood vessels in MOCK (**C**) and ZIKV infected (**D**) brain sectionat E15.5. Data presented here as the mean ± SEM from at least three sections prepared from three embryos obtained from three litters for each condition. (**E**) Quantification of the number of blood vessels in MOCK and ZIKV brains slice at E15.5. MOCK, n = 178 vessels; ZIKV, n = 188 vessels, Student’s t-test p > 0.05. **(F)** Quantification of the number of blood vessel branches and the branch length in μm(**G**). MOCK, n = 417 branches; ZIKV, n = 264 branches, Student’s t-test *p < 0.05 in F and ***p < 0.001 in G.(**H**) Quantification of the number of blood vessels, number of branches (**I**) or blood vessel branch length (J) in MOCK and ZIKV brains sections at P2. MOCK, n = 403 branches; ZIKV, n = 339 branches, Student’s t-test p > 0.05 in H and I, *p < 0.05 in J. (**K**) Immunohistochemisry of Laminin (red) stained blood vessels and Nestin (green) stained radial glia progenitors in MOCK (K) and ZIKV infected (**L**) in E15.5 brain sections (Scale bars = 50 µm). (**P**) Quantification of the Laminin and Nestin colocalization. There was a reduction of Laminin and Nestin colocalization (yellow labelling in K and L) in ZIKV infected brains compared to MOCK brains, Student’s t-test, *p < 0.05. (**O**) IB4 immunostaning in the VZ of ZIKV infected brains or MOCK brains (**M**). (**N**) Quantification of the blood vessel area in the VZ of E15.5 cortical sections. Student’s t-test **p < 0.01. Scale bars for A, B = 20 μm; C, D = 100 μm; K, L = 50 µm, and M,O = 10 μm.

**Figure 4:**
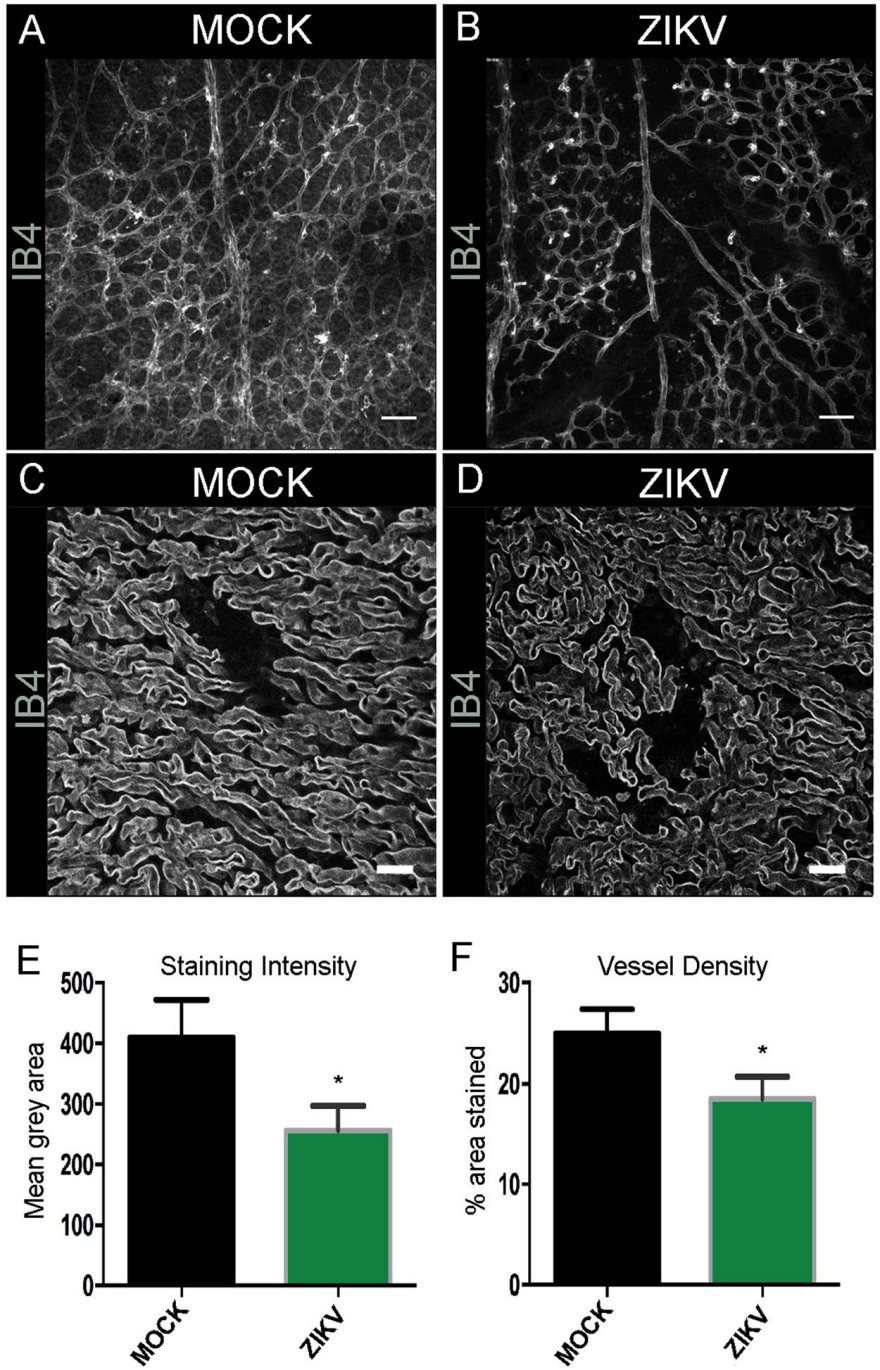
ZIKV infected placental vessel morphology at E15.5 Confocal images of IB4 stained wholemount retinas at P7 MOCK (**A**) and ZIKV infected animals (**B**). Scale bars = 100 μm. n = 7. Confocal images of IB4 stained blood vessels in 50 μm placenta section at E15.5 MOCK (**C**) and ZIKV infected animals (**D**). Scale bars = 20 μm. We analysed three sections from at least three different placentas for each condition. Blood vessel staining intensity (**E**) and vessel density (**F**) were measured with ImageJ and found reduced in ZIKV infected placenta compared to MOCK at E15.5. Student’s t-test *p < 0.05.

To examine the effect of ZIKV in blood vessel development in the cerebral cortex, we infected E12.5 pregnant females and harvested embryos three days after infection. We used IB4 staining to assess the vasculature morphology, comparing MOCK and infected brains at E15.5. ZIKV was detected by immunohistochemistry for 4G2 flavivirus envelope protein in the embryonic cerebral cortex at the proliferative zones and endothelial cells (Fig. 3A,B and Supplementary Fig. 1A,B). The vascular analysis of skeletonised IB4 stained blood vessels showed no overall change in the blood vessel number per field (875 × 375 µm^2^) within ZIKV infected cerebral cortex compared to MOCK (Fig. 3C,E). However, ZIKV infected brains displayed a significant decrease in the number of branches (Fig. 3F) and branch length (p = 0.0004; ZIKV −14.3 µm ± 0.9 µm, n = 3; MOCK −26.3 µm ± 0.7 µm, n = 3; Fig. 3G). At P2, the number of vessels and branches was not significantly different between ZIKV infected and MOCK brains (Fig. 3H,I). In contrast, at P2 there was still a significant (p = 0.03) reduction in branch length between MOCK (268.1 μm ± 0.013, n = 3) and ZIKV infected brains (219.7 μm ± 0.005, n = 3, Fig. 3J). To assess how much of the cortex was covered by vasculature at E15.5, we quantified the percentage of cortical tissue stained by IB4. Interestingly, there was a significant reduction in the proportion of vascular staining in the ventricular zone (quantified as vessel area, %; p = 0.005; Fig. 3M,O,P). Accordingly, using laminin to label blood vessels and nestin to label cortical progenitors, we observed a reduction of the co-localization of these markers in ZIKV brains compared to MOCK ones (Fig. 3K,L,P). To analyse the effect of infection with ZIKV on blood vessels in other tissues impaired by ZIKV, we collected and flattened retinas from P7 eyes. The pattern of vasculature displayed a similar reduction in vasculature as in cortex (Fig. 4A,B). We also examined E15.5 placenta three days after infection and stained blood vessels with IB4. Similarly, ZIKV was co-localized with the endothelial cells (Supplementary Fig. 1C,D) and the analysis of IB4, laminin and CD31 revealed a reduction of vessels’ intensity and density in the placenta tissue (Fig. 4C,F and Supplementary Fig.E-H).

### Angiogenic proteins are deregulated by congenital infection with ZIKV

**Figure 5:**
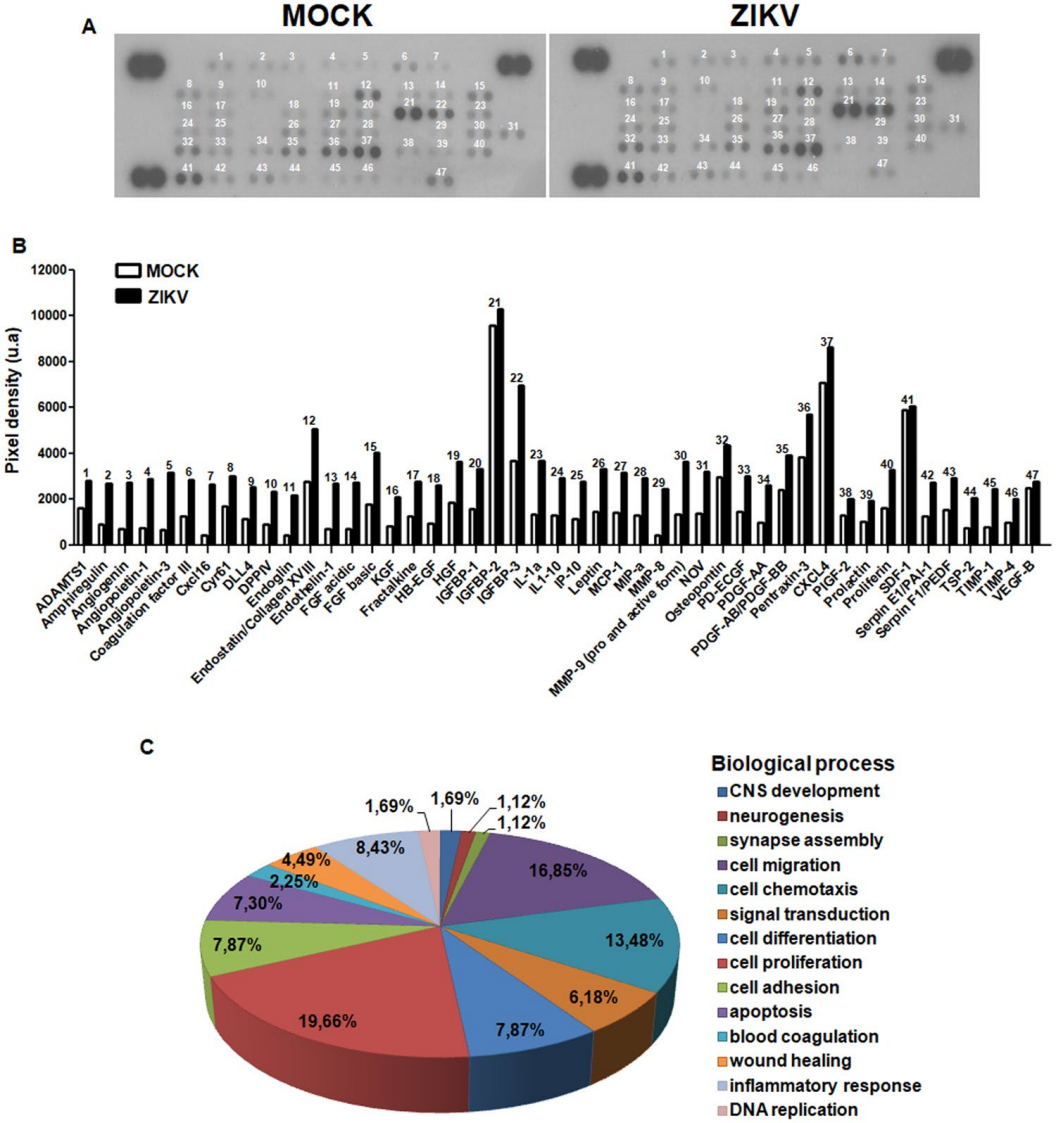
ZIKV infection alters cerebral cortex angiogenesis proteins levels. Cerebral cortex tissue extracts from three E15.5 embryos from three different litters of each experimental group were evaluated by angiogenesis proteomic profile. From 53 possible angiogenesis-related proteins, we identified 47 both present in MOCK and ZIKV infected brains (**A**). ZIKV infection increased the levels of 45 molecules (**B**). Besides their previously described role on angiogenesis, many identified proteins also contribute to other biological processes (**C**), including neurogenesis (19.66%), cell migration (16.85%), CNS development (7.87%) and inflammatory responses (8.43%).

The preciding data suggest that blood vessel development is perturbed by ZIKV infection. Since this process is regulated by multiple molecules, we performed a proteomics array for angiogenic related proteins. From 53 tested proteins, we identified 47 present in both MOCK and ZIKV infected cerebral cortex, although at different levels. In fact, ZIKV infection increased the expression of 45 angiogenic related molecules (Fig. 5A,B). Some of these proteins were previously described as having additional roles in brain development (Fig. 5C) such as neurogenesis, including Serpinf1 and DLL4^15,16^. Overall, 20% of the altered proteins have at least a partial role in neurogenesis. However, genes involved in angiogenic processes, which include cell differentiation, migration, chemotaxis, and adhesion combine together to represent approximately 45% of the altered genes. Altogether, this data suggests that congenital infection of ZIKV deregulates the balance of trophic and regulatory proteins that could contribute to the disruption of neurogenesis and angiogenesis in the developing cerebral cortex.

## Discussion

This study characterizes a new model of ZIKV-induced microcephaly like syndrome in mouse and identifies changes in the vasculature as part of the disease aetiology. We demonstrated that ZIKV infection at E12.5 in *Ifng*^*−/−*^ mice causes a reduction in brain size at P2. At E15.5 (3dpi), reductions in neurogenesis and area of both the progenitor zone and developing cortical plate were associated with a pattern of delayed vascular development.

Previous studies have demonstrated that direct ZIKV infection and death of the NPCs led to the reduced brain size^4,8^. Indeed, microcephaly can result from defective neurogenesis, an essential part of cortical development. However, cortical development is a complex phenomenon, which is regulated by a multitude of parallel processes, signalling factors, and extrinsic factors all occurring in tightly defined timescales. Therefore, the dysregulation of any of these processes could result in defective neurogenesis leading to cortical malformation. Cortical vascular plexus development can start as early as E11 in the mouse and is essential for the delivery of nutrients and regulating the expression of signalling molecules, which has an impact on neurogenesis^10,11^. The cortical blood supply develops from two plexi, the ventricular plexus and the pial plexus^12,13^. This study, along with previous research^17^, showed that ZIKV also infects the endothelial cells of the cortical vasculature in addition to neural progenitors. In particular, the vascular structure is abnormal in ZIKV infected mouse brains, especially in the ventricular zone, where there is also evidence of reduction of neuronal proliferation. The blood-brain barrier, specialisation of the cerebral endothelial cells that limit paracellular diffusion and transcellular transport, have been suggested to regulate the entry of most flaviviruses^18^. However, blood vessels are the pathway of ZIKV entrance into the brain parenchyma^18,19^, suggesting that the virus might have direct effects on the vasculature as well as other neural tissues. Some disruption of the blood-brain barrier has also been reported following ZIKV infection^20^, which may facilitate viral entry. However, the barrier changes described to date have not been quantified and were only described in a model of pathogenesis with substantial lethality in the postnatal period. Mladinish and colleagues^18^ suggest that in their primary human brain endothelial cells, ZIKV does not result in death of the cells or altered permeability. Instead, endothelial cells appear to act as a reservoir for viral replication, as well as contributing to inflammatory signaling following infection.

We found that following ZIKV injection, there was a general increase of the levels of angiogenic protein (with a few notable exceptions, such as CXCL4, VEGF), within the developing cortex. During angiogenesis, the balance of these different molecules is of particular importance, and in this context, there is a clear interplay between ‘normal’ and ‘abnormal’ growth and stabilisation signals. In the MOCK animals, there is a high concentration of VEGF and PDGF-BB proteins in the brain, which would promote endothelial cell proliferation and the recruitment of endothelial precursor cells and pericytes to the extending vascular network^21,22^. Following ZIKV injection, however, there is a substantial increase in Ang1, Ang3 and, to a lesser extent, endostatin and PEDF, which all suggest an anti-angiogenic status^22^. The exact effects and contribution of each of these proteins will need to be further elucidated. For instance, combined VEGF and Ang2 have a different effect on the development of vascular networks than either one individually^23^. The changes in protein concentration observed at E15.5 are likely to be a combination of the response of the blood vessels to ZIKV infection, as inflammation is a key regulator of angiogenesis^24^, and of the oxygen levels of the tissue.

Dividing progenitors are found in close proximity to blood vessels in the SVZ, and other neurovascular niches in the brain^13,25,26^. Changes in vascular development can result in altered neural patterning^12^ and, in addition, endothelial cells produce trophic factors which have important roles in neurogenesis^27–30^. While there is no dispute that ZIKV has a direct impact on human NPCs in the brain^3,8,19^, our study shows that a vascular defect may also contribute to ZIKV induced microcephaly. ZIKV infection of the endothelial cells may induce cortical vasculature abnormalities, which may further add to the abnormal neurogenesis.

In our murine model of ZIKV infection, we observed a more severe vascular developmental alteration at E15.5 that was much less prominent by P2, in contrast to the microcephaly-like phenotype, that was strongest at P2. In spite of this recovery, there was still a reduced vessel branch length present at P2 in ZIKV infected brains at birth. This suggests that ZIKV congenital infection promotes an acute insult that delays the vascular development during critical stages of neurogenesis that was partially recovered by birth. Such a delayed developmental progress is common in developmental disorders^31^, where the long-term consequences of the early disruption continue more subtly. The alterations in the vasculature in the cortical germinal zone might be just the “tip of the iceberg” in the ZIKV congenital infection. Our observations on the altered vascular development in the placenta and the retina suggest that ZIKV congenital infection might have a very general effect on the developing embryonic vasculature. It is important to note that the placenta produces several proinflammatory cytokines and expresses cytokine receptors. Several insults, such as LPS, bacterial and viral infection, including ZIKV, induce cytokine expression in the placenta impacting the fetus^32,33^.

The mouse model used in this study was produced by infection of ZIKV (10^5^ PFU), injected intravenously (IV) into the *Ifng*^*−/−*^ pregnant dam. In previous studies, a variety of ZIKV titres within the range of 10^3^–10^12^ PFU administered through different methods of systemic administration were employed to generate mouse models for ZIKV infection^34^, but these methods have often led to death of the young mice or whole body size reduction^8,35–38^. Here, the mouse model produced pups with smaller brains mimicking microcephaly-like phenotype that survived birth, thus enabling the opportunity to perform postnatal studies. Our results indicate that in the *Ifng*^*−/−*^ model, the embryos are not affected homogeneously by congenital ZIKV infection. Using PCR to detect viral loads in pup brains, we found that only some infected specimens were negative. Similarly, some morphological outcomes, such as skull size, also showed heterogeneous results. This non-uniform response to ZIKV infection in the production of microcephaly-like phenotypes has been reported by other authors, especially in models where the animals were not completely immunological deficient^7,39^. Therefore, *Ifng*^*−/−*^ mice seem to be appropriate to model an effective but also realistic ZIKV congenital infection. It would be interesting to investigate whether *Ifn* gene polymorphism resulting in lower cytokine levels in humans is associated with an increased prevalence of ZIKV induced microcephaly. Clustering patterns of microcephaly following ZIKV infection in Brazil hints at the possible role of environmental and socio-economic issues like availability of pregnancy supplements, age at pregnancy, vaccinations, and sanitation^40^. Our study suggests that ZIKV may cause additional angiogenic defects that could contribute to the reduced neurogenesis in the ventricular zone and the later reduced cortical volume. While the link between ZIKV infection, disruption of vascular development and reduced proliferation of neural progenitors needs to be further investigated, this is a novel mechanism that has a potential for direct therapeutic targeting.

## Materials and Methods

### Animal Model and Tissue Preparation

All methods, protocols, and procedures are in accordance with the relevant guidelines and regulations and were approved by the institutional research committee of the Federal University of Rio de Janeiro, Brazil, under the protocol #01200.001568/2013-87. Interferon gamma knockout (*Ifng*^*−/−*^) mice in the C57BL/6 background were obtained from the University of São Paulo (USP). *Ifng*^*−/−*^ pregnant mice were injected with ZIKV strain 766/AfZIKV 10^5^ plaque-forming units (PFU). Control *Ifng*^*−/−*^ pregnant mice were injected with supernatant of Vero cells (MOCK). ZIKV injections were performed at a retro-orbital position intraveneously (IV) at embryonic day (E) 12.5. All animals were homozygous and culled using cervical dislocation (adults) or decapitation (embryos and pups). Samples were collected from embryos at three-days post-infection (dpi, E15.5) or from pups at the 2^nd^ postnatal day (P2) and fixed by immersion in 4% paraformaldehyde (PFA, Sigma-Aldrich, USA) for 72 hours, then washed in phosphate-buffered saline (PBS).

### Assessment of brain size and weight

Images of whole brains obtained from three litters, n = 12 (3 mocks and 9 ZIKV), were taken with a light microscope (Leica, DFC490) from a dorsal view. Length and width of the cortex were measured (in pixels) using ImageJ at the longest (rostro-caudal) and widest points of the cortex (Fig. 1A,B). To compare changes in brain mass, whole brains were weighed (µg) on standard laboratory scales (AND, HM-120). To better characterize microcephaly-like phenotype in this animal model, we also performed micro computed tomography (mCT) of the skulls at P2. Images were obtained using a Bruker SkyScan1173 mCT scanner with the following parameters: isotropic voxel size of 8.55 µm, 30 kV and 180 µA. From these images, three dimensional reconstructions were built using Amira software, and the position of a set of 42 bilateral anatomical points (landmarks) was registered in each specimen as described by Gonzalez *et al*.^41,42^. From these registered landmarks we obtained comprehensive measures of skull size (centroid size) as defined by Bookstein^43^.

### Tissue Preparation for Fluorescent Immunohistochemistry

Brains and placenta for immunohistochemistry were embedded in 5% agarose (Bioline), in 0.1 M PBS, cut coronally at 50 µm with a vibrating microtome (VT1000S, Leica, Germany), and stored in 0.01% PBS-azide. At least three brains or placentas from either MOCK or ZIKV-infected groups from different litters, and three sections per brain or placenta were used for all immunohistochemistry experiments. Sections were washed in 0.1M PBS, incubated in blocking solution (2 hours, 2% donkey serum (Sigma-Aldrich) and 0.2% Triton-X100 (BDH) in 0.1M PBS), and then primary antibodies (rat anti-CTIP2, Abcam ab18465; rabbit anti-Pax6, Biolegend 901301; rabbit anti-pH3, Upstate 06-570; mouse anti-flavivirus group antigen, Millipore MAB10216) at 4 °C overnight. Sections were washed and incubated with fluorescent-conjugated secondary antibody (2 hours, either donkey anti-rabbit AlexaFluor568, Invitrogen A21206; donkey anti-mouse AlexaFluor647, Invitrogen A31571; donkey anti-goat AlexaFluor488, Molecular Probes, A-11055; or donkey anti-rat AlexaFluor488, Invitrogen A21208 at 1:500 dilution) and stained with 4′-6-diamino-2-phenylindole (DAPI, Invitrogen, 1:1000 dilution in 0.1M PBS) nuclear counterstain. Retinas were removed at P7, flattened and fixed with cold (−20 °C) methanol. Free-floating retina, brain and placenta sections stained with Isolectin B4 (IB4, Vector, FL1201, 1:200) were incubated with the stain for 2 hours before DAPI counterstain.

### Imaging, quantifications and Statistical Analysis

Fluorescently labelled sections were imaged using a fluorescence microscope (Leica, DFC500) and a confocal laser-scanning microscope (LSM710, Zeiss). ImageJ was used to determine the area (in pixel units) covered by ventricular and subventricular zones and cortical layers on fluorescence microscopy images of DAPI counterstained samples. Pax6 and Ctip2 immunohistochemistry were used to define the radial glial cells and newly emerging cell layers of the cortical plate, and the numbers of immunoreactive cells were quantified. Pax6+, Ctip2+ and pH3+ number of cells were counted in three sections of at least three different brains of each experimental group. We used the field of view of 20X for Pax6 and Ctip2 and 40X for Ph3 analysis. Analysis of the cortical vasculature was carried out in three sections of at least three different brains from different litters stained with IB4. Images were acquired using a confocal microscope as described above. To determine vessel characteristics, the threshold tool (ImageJ) was used to produce binary images, and images were de-noised (using the ImageJ ‘Despeckle’ tool) to remove excessive non-specific stain signal. The width of vessels (µm) was determined using the histogram tool to measure the x-axis distance between the margins of each vessel, perpendicular to the pial surface. Vessel area was determined by the proportion of vascular staining within each cortical region. Skeletonised structure of the blood vessels was obtained using the ImageJ plugin ‘skeletonise’, to quantify vessel number, branch number, and branch length. All data were analysed with Excel or GraphPad Prism. Statistical evaluation was performed by Student’s unpaired t-test. Data in graphs are presented as mean ± SEM. *p ≤ 0.05, **p ≤ 0.01, ***p ≤ 0.001, ****p ≤ 0.0001.

### Proteome profile array

Cerebral cortex tissue extracts from E15.5 MOCK or ZIKV infected embryos were processed for analysis of angiogenesis-related proteins levels by using the Proteome Profiler™ Mouse Angiogenesis Antibody Array kit (R&D Systems) that identifies 53 angiogenesis-related proteins. Proteome assay and analysis were performed according to manufacturer’s instructions.

### Viral detection

RNA was extracted from brain, eye and spleen tissue samples using RNeasy Plus Mini Kit (QIAgen), following the manufacturer’s recommendations. Viral RNA was quantified using One Step Taqman RT-qPCR (Thermo Fisher Scientific) on a 7500 Real-Time PCR System (Applied Biosystems) with primers and probe designed by Lanciotti and collaborators^44^. ZIKV copies were calculated by interpolation onto an internal standard curve formed of eight 10-fold serial dilutions of a synthetic ZIKV RNA based on the Asian lineage. The ZIKV quantification was displayed in ZIKV RNA copies per gram of tissue.

## Electronic supplementary material

Supplementary Figure 1
