# Supplementary material for: Zika virus impairs the development of blood vessels in a mouse model of congenital infection
Source: Sci Rep. 2018 Aug 24;8:12774. doi: 10.1038/s41598-018-31149-3 (PMC6109170; doi:10.1038/s41598-018-31149-3)
Supplement: Supplementary file 1 — Supplementary Figure 1 [file 41598_2018_31149_MOESM1_ESM.docx]

**Supplementary Material**

**Zika virus impairs the development of blood vessels in a mouse model of congenital infection**

Garcez, P.P.^1,2*@^, Stolp, H.B.^2,3*@^, Sravanam, S.^2^, Christoff, R.R.^1^, Ferreira J.C.C.G.^1^., Dias, A.A.^4^, Pezzuto P.^5^, Higa L.M.^5^, Barbeito-Andrés, J^1^., Ferreira R.O.^1^, Vieira de Andrade C.C.B.^6^., Siqueira M^1^., Santos, T.M.P.^7^, Drumond, J.^1^, Hoerder-Suabedissen, A.^2^, Victorino, C.^8^, Tovar-Moll, F.^1,8^, Lopes R.T.^7^, Fragel-Madeira L^9^., Lent, R.^1^, Ortiga-Carvalho T.M.^6^, Stipursky J.^1^, Bellio, M.^4^, Tanuri, A.^5^, Molnár, Z.^2@^

**
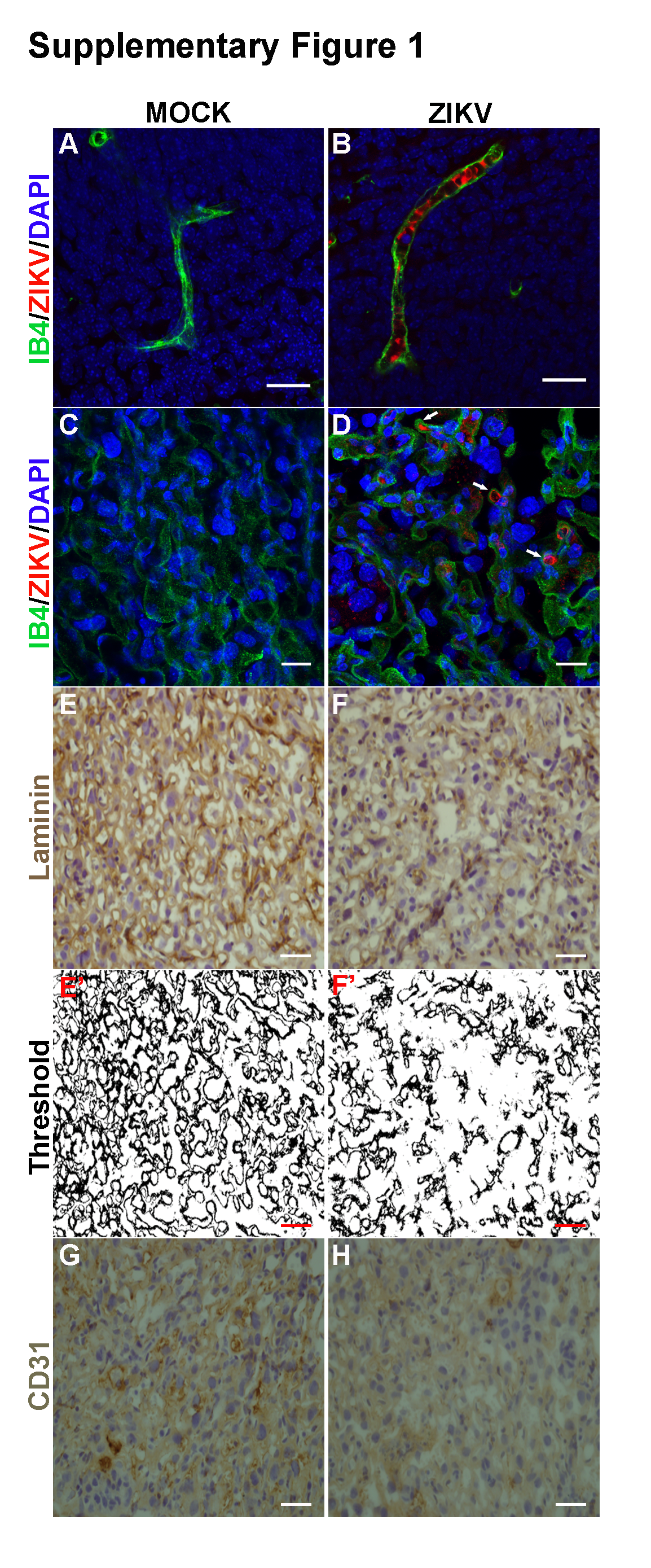
**

**Supplementary Figure 1: ZIKV infected blood vessels at E15.5**

Confocal images of IB4 (green) stainning and 4G2 (red) immunehistochemistry in 50μm cerebral cortex section at E15.5 MOCK (**A**) and ZIKV infected animals (**B**). Confocal images of IB4 (green) stainning and 4G2 (red) immunehistochemistry in 50μm placenta section at E15.5 MOCK (**C**) and ZIKV infected animals (**D**). Arrows point to infected cells. We analysed three sections from at least three different placentas for each condition. Confocal images of laminin immunohistochemistry in 50μm placenta section at E15.5 MOCK (**E**) and ZIKV infected animals (**F**). (E´) and (F´) are binary representations of E and F using a ImageJ threshold tool. Confocal images of CD31 immunohistochemistry in 50μm placenta section at E15.5 MOCK (**G**) and ZIKV infected animals (**H**). Scale bars in A-D=20μm and E-H=40μm.
